# Novel Catabolic Pathway of Quercetin-3-*O*-Rutinose-7-*O*-α-L-Rhamnoside by *Lactobacillus plantarum* GDMCC 1.140: The Direct Fission of C-Ring

**DOI:** 10.3389/fnut.2022.849439

**Published:** 2022-03-16

**Authors:** Guitao Huang, Mingwen Lai, Canhua Xu, Shan He, Lihong Dong, Fei Huang, Ruifen Zhang, David James Young, Hesheng Liu, Dongxiao Su

**Affiliations:** ^1^School of Chemistry and Chemical Engineering, Guangzhou University, Guangzhou, China; ^2^Sericultural & Agri-Food Research Institute, Guangdong Academy of Agricultural Sciences/Key Laboratory of Functional Foods, Ministry of Agriculture and Rural Affairs/Guangdong Key Laboratory of Agricultural Products Processing, Guangzhou, China; ^3^College of Engineering, Information Technology & Environment, Charles Darwin University, Darwin, NT, Australia; ^4^Zhejiang Provincial Top Discipline of Biological Engineering (Level A), Zhejiang Wanli University, Ningbo, China

**Keywords:** *Bacteroides uniformis*, *Lactobacillus*, lychee, gut microbiota, C-ring fission

## Abstract

Lychee pulp phenolics (LPP) is mainly catabolized in the host colon, increasing the abundances of *Bacteroides* and *Lactobacillus*. Herein, five selected gut microbial strains (*Bacteroides uniformis, B. thetaiotaomicron, Lactobacillus rhamnosus, L. plantarum*, and *L. acidophilus*) were separately incubated with LPP to ascertain the specific strains participating in phenolic metabolism and the corresponding metabolites. The results indicated that *B. uniformis, L. rhamnosus*, and *L. plantarum* were involved in LPP utilization, contributing to 52.37, 28.33, and 45.11% of LPP degradation after 48 h fermentation, respectively. Unprecedentedly, the metabolic pathway of the major phenolic compound quercetin-3-*O*-rutinose-7-*O*-α-L-rhamnoside by *L. plantarum*, appeared to be the direct fission of C-ring at C2–O1 and C3–C4 bonds, which was proved from the occurrence of two substances with the deprotonated molecule [M–H]^−^ ion at *m/z* 299 and 459, respectively. Meanwhile, it was fully confirmed that *B. uniformis* participated in the catabolism of isorhamnetin glycoside and procyanidin B2. In the *B. uniformis* culture, kaempferol was synthesized through dehydroxylation of quercetin which could be catabolized into alphitonin by *L. rhamnosus*. Furthermore, LPP metabolites exerted higher antioxidant activity than their precursors and gave clues to understand the interindividual differences for phenolic metabolism by gut microbiota.

## Introduction

Lychee (*Litchi chinensis* Sonn.), a subtropical to tropical fruit with significant nutrient value, was originally grown in the southern China and northern Vietnam but has now spread to over twenty countries around the world (1). Lychee pulp possesses a great diversity of bioactivities, including but not limited to antioxidant, hypolipidemic, and anti-inflammatory activities (2, 3). Because of its various benefits to human health, lychee pulp was added to the list of functional food in 2012 by the U.S. Department of Agriculture (4). The health benefits of lychee pulp have been attributed to its abundant nutritional components, among which phenolic compounds are generally considered the pivotal ones (1). Previous studies have demonstrated that lychee pulp is abundant in phenolic compounds, including a large amount of flavonoids (e.g., quercetin-3-*O*-rutinose-7-*O*-α-L-rhamnoside, rutin, and procyanidin B2) and trace amounts of phenolic acids (5). It has also been reported that lychee pulp phenolics (LPP) is soluble and stable in the gastrointestinal environment (6). However, recent research has revealed that phenolic-enriched lychee pulp extracts could not be transformed and absorbed by intestinal epithelial cells with phase-I or phase-II enzymes (7), but instead passed through to the colon. Notably, our previous work showed that few to no phenolics were detected in the fecal extracts of mice after LPP supplementation for 21 days, suggesting that LPP was catabolized in the colon (6). Rutin is one of the main components of LPP, second only to quercetin-3-*O*-rutinose-7-*O*-α-L-rhamnose (QRR). The microbial metabolites of rutin have been identified previously (8), while those of the other main phenolics (including QRR and procyanidin B2) of lychee pulp remain unknown.

The human colon is a highly complex environment where limited carbon sources (e.g., flavonoid glycosides) are fully utilized by numerous microbiota defined as the human gut microbiota (9). The intestinal microbiota have evolved to effectively metabolize exogenous substances such as plant-derived components (10), thus closely related to the catabolism of phenolic compounds in the human body. In the last few years, most of the researches regarding phenolics were focused on the regulatory effects of phenolics on gut microbiota or fecal bacterial flora (11). However, a recent study offered a new perspective, pointing out that different microbial species played a specific role in phenolic metabolism (12). It was also inferred that the microbial units that were enriched by phenolics supplementation participated in the phenolic metabolism. High-throughput sequencing data showed that the abundances of *Bacteroides* (especially for *B. uniformis* and *B. thetaiotaomicron*) and *Lactobacillus* in mice were effectively upregulated after LPP supplementation for 2 weeks (13). Therefore, it was reasonable to infer that the metabolism of LPP might be related to some microbial species referring to *Bacteroides* or *Lactobacillus*. Meanwhile, previous research has revealed that *Bacteroides* generates enzymes to degrade rhamnose-containing substances, including flavonoid rhamnose (9). *Lactobacillus* species (e.g., *L. rhamnosus, L. plantarum*, and *L. acidophilus*) were enriched in the host colon after flavonoids supplementation, also suggesting the interaction between *Lactobacillus* and flavonoids (14). Despite the advances in our knowledge of these phenolics–microbiota interactions, the identification of specific microbial strains that participate in the catabolism of LPP, and the corresponding products remain incomplete. Besides, coculture fermentation and fecal flora fermentation are difficult to ascertain the specific microbiota participating in phenolic metabolism. Whereas, the metabolic effects and action sites of the specific strains on phenolic compounds could be clarified in individual fermentation.

Consequently, in this study, five gut microbial strains, namely, *B. uniformis, B. thetaiotaomicron, L. rhamnosus, L. plantarum*, and *L. acidophilus*, were incubated with LPP, respectively, in an effort to identify and quantify the metabolites of dominant phenolics in LPP and to further explore both the identical and unique pathways of phenolic catabolism by gut microbe.

## Methods

### Materials and Chemicals

Fresh ripe lychee (cv. *Guiwei*) was purchased from a fruit market in Guangzhou, China. Quercetin-3-*O*-rutinose-7-*O*-α-L-rhamnoside was prepared following a previous method reported by our team (5). Other phenolic standards and all solvents for chromatographic analysis, were purchased from Sigma-Aldrich Chemical Corporation (Oakville, Ontario, Canada).

### Preparation of LPP Solution

The preparation of LPP was achieved by a previously reported procedure (2). Briefly, lychee pulp (50 g) was mashed with 80% aqueous ethanol (150 ml) in a Philips blender for 5 min. After centrifuged at 4,000 × *g* for 10 min, the supernatant was collected and the residue re-extracted (three times). The pooled supernatant was evaporated under vacuum at 40°C and added onto an HPD-826 resin column (Cangzhou Bonchem Corporation Ltd., Cangzhou, China). The column was washed with ultrapure water, and then the organic phase acquired by eluting with 95% aqueous ethanol (*v*/*v*) was collected, rotary evaporated and lyophilized (Biosafer-10B) to obtain LPP. Before fermentation, LPP solution (10 mg/ml, prepared by dissolving 0.5 g LPP in phosphate-buffered saline (PBS) to make 50 ml) was pasteurized at 65°C for 30 min on water bath.

### Microbial Strains and Cultures

The microbial strains (*Bacteroides uniformis* GDMCC 1.898, *Bacteroides thetaiotaomicron* GDMCC 1.1104, *Lactobacillus rhamnosus* GDMCC 1.1798, *Lactobacillus plantarum* GDMCC 1.140 and *Lactobacillus acidophilus* GIM 1.67) were purchased from Guangdong Microbial Culture Collection Center, China. The modified Schaedler broth containing tryptone soy broth (10 g/l), casein pancreatic peptone (2.43 g/l), soy peptone (0.43 g/l), meat extract (2.15 g/l), yeast extract (5 g/l), glucose (5 g/l), Tris-HCl (0.75 g/l), L-cysteine (0.4 g/l), hemin (0.01 g/l), and vitamin K3 (0.5 mg/l) was prepared and sterilized following the reported procedure by Benitez-Paez et al. (15) *Bacteroides. spp* were incubated (1%) in modified Schaedler broth at 37°C for 96 h. *Lactobacillus. spp* were incubated (1%) in MRS broth at 37°C for 48 h. Incubations were carried out under anaerobic condition (10 H_2_, 10 CO_2_, and 80% N_2_).

### *In-vitro* Fermentation of LPP by Single Microbial Strains

After twice activation, microbial cells were harvested by centrifugation (8,000 × *g* for 10 min at 4°C), washed twice and resuspended in sterile PBS to obtain bacterial suspension at the final concentration of 9.0 Log (CFU/ml).

The basal medium was prepared following the protocol reported by Cardenas-Castro et al. (16). The basal medium containing peptone (2 g/l), yeast extract (2 g/l), NaCl (0.1 g/l), K_2_HPO_4_ (0.04 g/l), KH_2_PO_4_ (0.04 g/l), MgSO_4_·7H_2_O (0.01 g/l), CaCl_2_·2H_2_O (0.01 g/l), NaHCO_3_ (0.01 g/l), cysteine HCl (0.5 g/l), bile salts (0.5 g/l), Tween 80 (2 ml/l), and 0.2 g hematin (diluted in 5 ml of NaOH) was adjusted to pH = 7.0 ± 0.2 and autoclaved at 121°C for 15 min.

**For the LPP group**, 1 ml of the pasteurized LPP solution (10 mg/ml) was mixed with 8 ml of sterile basal nutrient medium and 1 ml of each bacterial suspension in a 15 ml tube in order to reach 7.0–8.0 Log (CFU/ml). **For the blank control (BLK) group**, sterile PBS (1 ml) was mixed with 8 ml of sterile basal nutrient medium and 1 ml of each bacterial suspension in tube, as a blank control. All the tubes were incubated at 37°C under anaerobic condition. Cultures were taken out for analysis at different time points (0, 12, 24, 36, and 48 h). Each fermentation process was conducted independently in triplicates.

### Determination of Turbidity and pH Values

The turbidity of each culture was quantified by measuring optical density at 600 nm in a microplate reader (TECAN Infinite 200, TECAN, Switzerland). The pH change was determined by a PB-10 pH meter (Hetian Apparatus Corporation, Shanghai, China) immediately after the cultures were removed from the anaerobic incubator.

### Enumeration of Bacteria

The viable cell counts of cultures before and after fermentation (0 and 48 h) were quantified by plate count. The samples were diluted with sterile normal saline until 10^6^-10^9^ dilutions. Aliquots of dilutions were plated in triplicate on Schaedler anaerobe agar (Oxoid) for *Bacteroides* strains, while on MRS agar for *Lactobacillus* strains (15, 17). Plates were incubated at 37°C under anaerobic condition for 48 h.

### Samples Preparation for Phenolics and Metabolites Analysis

All the cultures were centrifuged at 8,000 × *g* for 15 min at 4°C. Then, the supernatants of cultures were harvested and defined as the **supernatant** sample. Aliquots of each **supernatant** sample were filtered through 0.22 μm membrane and then subjected to chromatographic analysis.

The residues of cultures were mixed with methanol (4 ml) and then subjected to ultrasonication for 10 min. The obtained mixtures were centrifuged at 8,000 × *g* for 15 min at 4 C and the methanol fraction was collected and defined as the **residue** sample. Aliquots of **residue** sample were filtered through 0.22 μm membrane and stored at −80°C until analysis.

### Quantification of Individual Phenolic Compounds by HPLC-DAD

Individual phenolic contents in the supernatant and residue of cultures were quantified using the Agilent 1260 HPLC System equipped with a diode array detector (DAD), as reported previously (5). Chromatographic separation was performed on the Zorbax SB-C18 columns (250 × 4.6 mm, 5 μm, Agilent) at 30°C. The parameters were: flow rate, 1.0 ml/min and injection volume, 20 μl. Mobile phase comprised 0.4% glacial acetic acid (solvent A) and acetonitrile (solvent B). The gradient elution program was applied as follows: 0–40 min, 5–25% B; 40–45 min, 25–35% B; and 45–50 min, 35–50% B. Based on retention times measured for the authentic standards, the identification of peaks detected at 280 nm was confirmed. Compounds were quantified with the standard curves established by the corresponding phenolic substances, according to our previous work (13). Results were expressed as mg per gram dry weight (DW) of LPP.

### Identification of Tentative Phenolics and Metabolites of LPP by UHPLC-ESI-QTOF-MS/MS

Compared with the phenolics contents in the supernatant of initial cultures (0 h), the supernatants of cultures with significant change (*p* < 0.05) in phenolics contents after fermentation for 48 h, were subjected for qualitative analysis and follow-up analysis.

Compound identification was achieved by UHPLC-ESI-QTOF-MS/MS as reported previously by our team (18). The analysis was performed with an Agilent 1290 UHPLC system coupled to a Triple-TOF 5600+ mass spectrometer (Agilent Technologies, California, USA) and equipped with an Agilent EclipsePlus C18 column (2.1 × 100 mm, 1.8 μm, Palo Alto, California, USA) and ESI source operating in negative ionization mode. The parameters set were: capillary voltage, 4,500 V; flow rate, 0.4 ml/min; column temperature 35°C; ion source temperature 500°C; and injection volume, 4 μl. The mobile phase consisted of 0.4% formic acid in water (A) and acetonitrile (B). The analysis was carried out with a gradient elution as follows: 0–16 min, 5–25% B; 16–18 min, 25–35% B; 18–20 min, 35–50% B. The abundance of ions (100–1,000 *m/z*) was scanned. Confirmation was obtained by comparison with external standards whenever available and by consulting the phytochemical dictionary of natural products database (DNP).

### Determination of Total Phenolic Contents

Total phenolic content (TPC) in the supernatant of each culture was determined as reported previously (6). The TPC was detected using Folin–Ciocalteu reagent and presented as gallic acid equivalent (mg GAE/g DW).

### Quantification of Antioxidant Activity

Ferric-reducing antioxidant power (FRAP) was measured, as described previously (6). Briefly, 2.7 ml of FRAP working solution was reacted with 0.3 ml of sample in the dark for 10 min. The absorbance was then detected at 593 nm using a microplate reader. FRAP values were expressed as Trolox equivalents (mmol TE/g DW).

The ABTS^**.+**^ scavenging capacity was measured as described by Lv et al. (19). Briefly, 2.4 ml of ABTS^**.+**^ working solution (diluted in ethanol) was reacted with 0.6 ml of sample for 6 min. The absorbance of reactant was then measured at 734 nm. Results were expressed as Trolox equivalents (μmol TE/g DW).

### Statistical Analysis

Experimental data were expressed as the mean ± SD after triplicate experiments. One-way ANOVA with Tukey's test was applied to estimate between-group statistical difference using SPSS software version 24 (Chicago, Illinois, USA) and the significance level was established at *p* < 0.05.

## Results

### Effects of LPP on the Turbidity, pH Values, and Viable Cell Counts of Single Microbial Cultures

The turbidities of five microbial cultures were recorded and displayed in Supplementary Figure 1. For the same tested strain, the turbidities of cultures in the LPP group were higher than those in the BLK group before fermentation. After 48 h of fermentation with LPP, the OD_600_ values of all the cultures were significantly (*p* < 0.05) higher than their corresponding initial values. Notably, the OD_600_ value of *L. rhamnosus* cultures in the BLK group significantly (*p* < 0.05) decreased from 0.44 to 0.41, while a dramatic (*p* < 0.05) increase in the LPP group was noticed. Unlike other strains, the turbidity of *L. acidophilus* cultures in both the LPP and BLK groups did not reach a stable trend over 48 h of fermentation.

**Figure 1:**
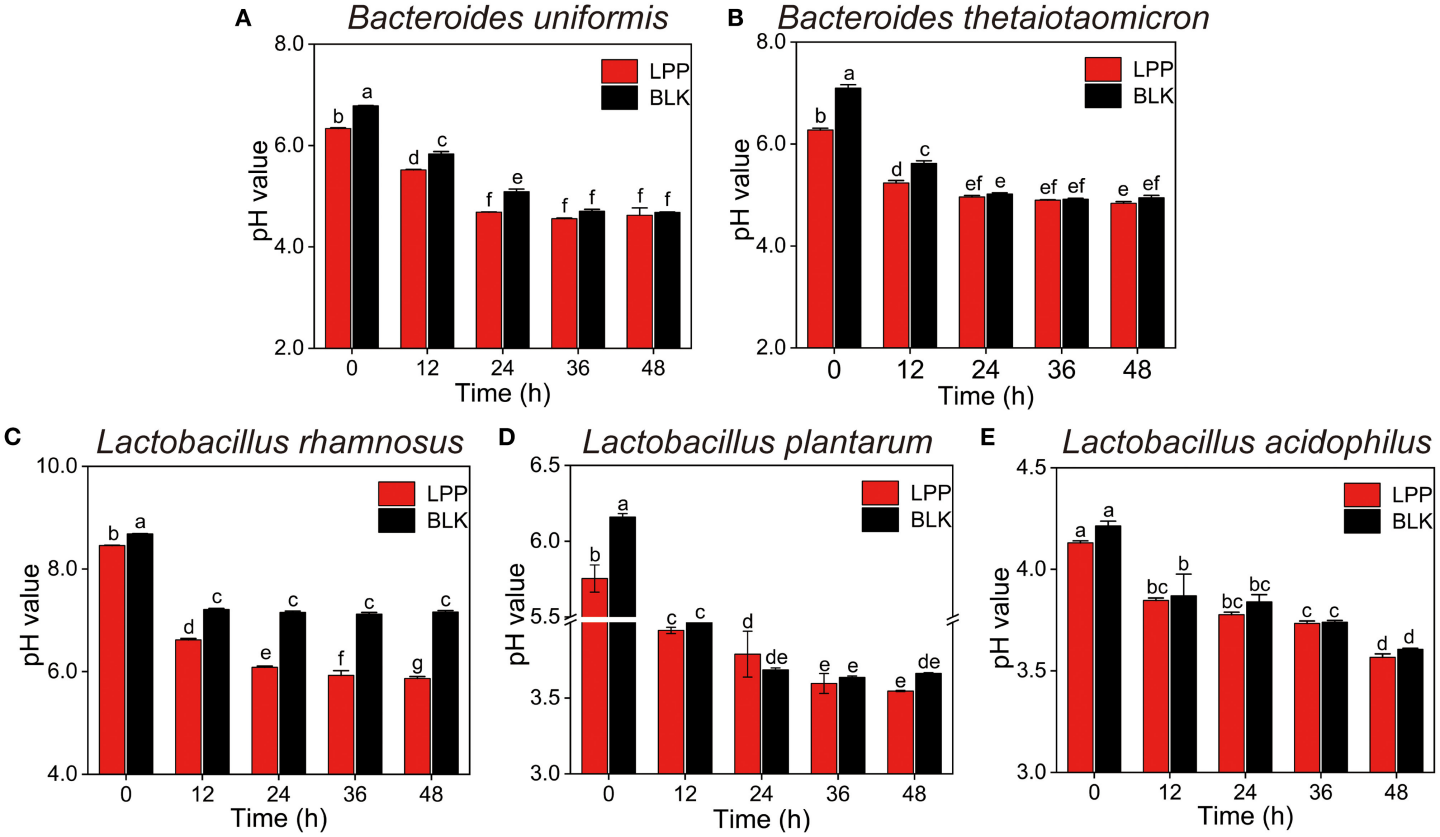
Changes in pH values of five microbial cultures during fermentation with lychee pulp phenolics (LPP). The BLK group was fermented with phosphate-buffered saline (PBS) solution. Bars with no letter in common are significantly different (*p* < 0.05). **(A)** *Bacteroides uniformis*, **(B)** *Bacteroides thetaiotaomicron*, **(C)** *Lactobacillus rhamnosus*, **(D)** *Lactobacillus plantarum*, and **(E)** *Lactobacillus acidophilus*.

The pH values of all the cultures displayed a dramatic (*p* < 0.05) descending trend during the whole fermentation (Figure 1). In comparison to the BLK group, the pH values of all the cultures at 0 h were significantly (*p* < 0.05) decreased after LPP supplementation. Notably, the pH values of *L. rhamnosus* culture in the BLK group exhibited a stable trend from hour 12 on. For *B. uniformis, B. thetaiotaomicron*, and *L. plantarum* cultures, the pH values of the BLK group were comparable to that of the LPP group from hour 36 on. The pH values of *L. acidophilus* cultures in neither the LPP nor BLK groups showed a stable trend after 48 h of fermentation.

**Figure 2:**
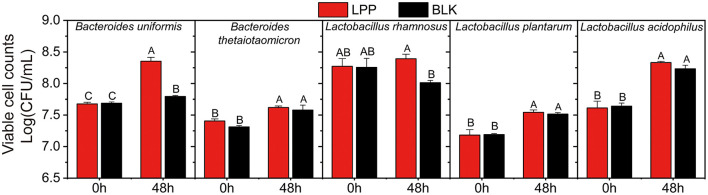
The viable cell counts of five gut microbial strains before and after fermentation (at 0 and 48 h) with LPP. BLK group was fermented with PBS solution. Bars with no letter in common are significantly different (*p* < 0.05).

To further reveal the proliferation-enhancing effects of LPP on gut microbiota, the viable cell counts of all the cultures before and after fermentation were carried out and displayed in Figure 2. For the same strain, the viable cell counts in the LPP and BLK groups showed no significant difference (*p* > 0.05) before fermentation. At 48 h of fermentation, the viable cell count of *B. uniformis* in the LPP group was 0.65 Log (CFU/ml) higher (*p* < 0.05) than that in the BLK group. As for *L. rhamnosus*, the viable cell count in the BLK group decreased from 8.26 to 8.01 Log (CFU/ml) during fermentation, while a slight augment was observed in the LPP group. As for the viable cell count of *B. thetaiotaomicron, L. plantarum*, and *L. acidophilus*, no significant difference (*p* > 0.05) was observed before and after fermentation between the LPP and BLK groups.

### Effects of Single Microbial Fermentation on the Contents of Individual Phenolics

**Figure 3:**
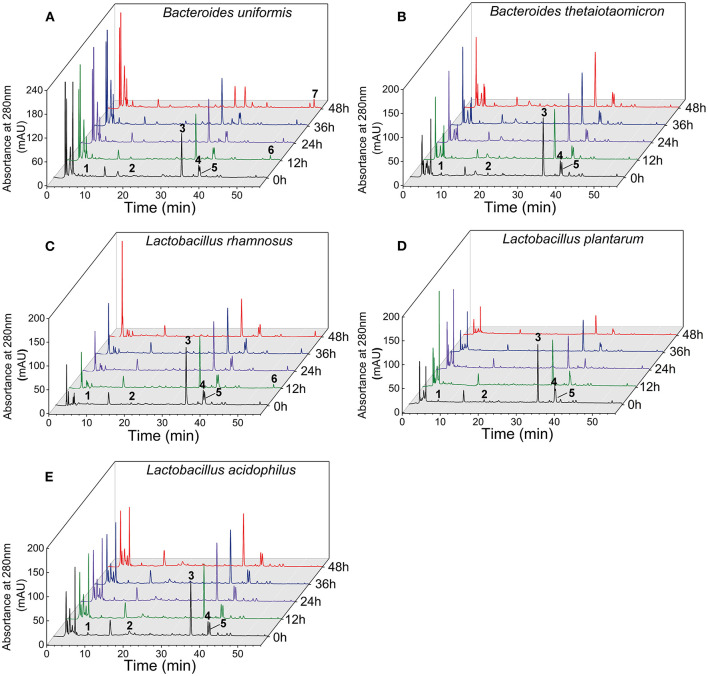
Chromatograms of the supernatants of five gut microbial cultures during fermentation with LPP at 280 nm. Peak 1, gallic acid; peak 2, (+)-catechin; peak 3, quercetin-3-*O*-rutinose-7-*O*-α-L-rhamnoside (QRR); peak 4, ferulic acid; peak 5, rutin; peak 6, quercetin; peak 7, kaempferol.

The contents of monomeric phenolics in the supernatant and residue of five cultures during the whole fermentation are given in Table 1 and Supplementary Table 1. In the supernatant of *B. uniformis, L. rhamnosus*, and *L. plantarum* culture, quercetin-3-*O*-rutinose-7-*O*-α-L-rhamnosise (QRR) was remarkably (*p* < 0.05) degraded by 55.87, 32.21, and 47.82%, respectively, over 48 h of fermentation. In contrast, the contents of QRR in the supernatants of *B. thetaiotaomicron* or *L. acidophilus* culture maintained a relatively stable level (*p* > 0.05) as compared with the initial values during the whole fermentation period. After 48 h of fermentation, the rutin contents in the supernatants decreased by 72.82, 16.05, and 22.87%, respectively, for *B. uniformis, L. rhamnosus*, and *L. plantarum* cultures. Specifically, during the fermentation with *L. rhamnosus* till 36 h, the increments in the contents of rutin in both the supernatant and residue were observed (Figure 3C and Supplementary Figure 2). Meanwhile, the contents of rutin in the supernatant of *L. rhamnosus* culture were significantly (*p* < 0.05) higher than the initial values after 36 h of fermentation. From hour 12 on, quercetin could be detected in the supernatants of *B. uniformis, L. rhamnosus*, and *L. plantarum* cultures.

**Table 1:** Phenolic contents of the supernatants of five microbial cultures during fermentation^a^.

**Groups**	**Compound (mg/g)**	**Fermentation times**				
		**0 h**	**12 h**	**24 h**	**36 h**	**48 h**
*B. uniformis*	QRR	142.14 ± 6.85^b^	145.93 ± 4.08^b^	136.59 ± 6.04^b^	136.13 ± 6.65^b^	62.73 ± 5.62^a^
	Rutin	9.16 ± 2.26^b^	14.37 ± 0.48^b^	12.62 ± 0.22^b^	12.39 ± 1.16^b^	2.49 ± 0.11^a^
	Quercetin	<LOQ	0.58 ± 0.01^a^	0.82 ± 0.01^a^	0.94 ± 0.11^a^	3.45 ± 0.65^b^
	(+)-Catechin	6.44 ± 0.69^b^	4.83 ± 0.46^a^^b^	4.62 ± 0.20^a^^b^	3.81 ± 0.90^a^	5.15 ± 0.19^a^^b^
	Gallic acid	3.55 ± 0.14^bc^	2.54 ± 0.54^a^^b^	3.79 ± 0.22^c^	2.52 ± 0.52^a^^b^	2.28 ± 0.32^a^
	Ferulic acid	5.64 ± 0.15^b^	5.89 ± 0.06^b^	5.77 ± 0.10^b^	5.77 ± 0.22^b^	3.42 ± 0.20^a^
	Total	166.94 ± 8.40^b^	174.13 ± 3.76^b^	164.21 ± 6.15^b^	161.55 ± 8.24^b^	79.51 ± 6.49^a^
*B. thetaiotaomicron*	QRR	150.48 ± 5.18^a^	141.04 ± 4.68^a^	143.80 ± 8.88^a^	148.65 ± 5.47^a^	151.31 ± 1.64^a^
	Rutin	15.80 ± 0.37^b^	12.32 ± 1.31^a^^b^	13.23 ± 1.69^a^^b^	13.75 ± 1.17^a^^b^	14.73 ± 0.12^b^
	Quercetin	<LOQ	<LOQ	<LOQ	<LOQ	<LOQ
	(+)-Catechin	7.06 ± 0.13^a^	6.91 ± 0.89^a^	7.45 ± 0.43^a^	8.31 ± 0.94^a^	7.47 ± 0.11^a^
	Gallic acid	2.61 ± 0.01^a^	3.06 ± 0.32^a^	2.81 ± 1.11^a^	2.64 ± 0.79^a^	3.14 ± 0.04^a^
	Ferulic acid	6.25 ± 0.03^b^	5.64 ± 0.06^a^	5.71 ± 0.29^a^	5.86 ± 0.19^a^^b^	6.08 ± 0.06^a^^b^
	Total	182.21 ± 5.74^a^	168.97 ± 7.27^a^	173.00 ± 12.41^a^	179.21 ± 8.50^a^	182.73 ± 1.83^a^
*L. rhamnosus*	QRR	156.45 ± 4.94^d^	140.76 ± 0.79^c^	129.39 ± 0.75^bc^	117.99 ± 1.33^b^	106.06 ± 2.6^a^
	Rutin	11.53 ± 0.97^a^^b^	12.55 ± 0.49^abc^	15.65 ± 0.80^bc^	16.04 ± 0.32^c^	9.68 ± 1.32^a^
	Quercetin	<LOQ	1.48 ± 0.01^a^	1.75 ± 0.00^b^	1.89 ± 0.01^b^	2.08 ± 0.06^c^
	(+)-Catechin	5.75 ± 0.04^a^	7.04 ± 0.04^d^	6.78 ± 0.04^c^	6.56 ± 0.03^b^	6.46 ± 0.05^b^
	Gallic acid	1.91 ± 0.01^a^	1.95 ± 0.00^a^	1.98 ± 0.00^a^	1.96 ± 0.01^a^	2.01 ± 0.00^a^
	Ferulic acid	6.29 ± 0.25^c^	5.49 ± 0.01^bc^	5.33 ± 0.05^bc^	5.12 ± 0.08^a^^b^	4.09 ± 0.39^a^
	Total	181.93 ± 4.27^d^	169.27 ± 0.35^c^	160.88 ± 1.55^c^	149.57 ± 0.94^b^	130.38 ± 1.01^a^
*L. plantarum*	QRR	141.04 ± 8.01^b^	127.48 ± 13.66^a^^b^	108.99 ± 17.01^a^^b^	109.22 ± 16.82^a^^b^	73.60 ± 14.86^a^
	Rutin	9.56 ± 0.19^b^	9.22 ± 1.45^b^	10.10 ± 0.59^b^	9.55 ± 0.21^b^	6.53 ± 1.09^a^
	Quercetin	<LOQ	1.56 ± 0.00^b^	1.63 ± 0.14^b^	1.43 ± 0.02^b^	1.10 ± 0.05^a^
	(+)-Catechin	7.66 ± 0.62^c^	6.93 ± 1.06^bc^	4.49 ± 0.45^a^^b^	4.41 ± 0.36^a^^b^	3.15 ± 0.40^a^
	Gallic acid	3.09 ± 0.01^a^^b^	3.65 ± 0.02^c^	3.45 ± 0.24^bc^	3.11 ± 0.01^a^^b^	2.74 ± 0.06^a^
	Ferulic acid	6.90 ± 0.01^b^	7.11 ± 0.09^b^	6.78 ± 0.44^b^	6.92 ± 0.01^b^	5.25 ± 0.31^a^
	Total	168.25 ± 8.41^bc^	155.96 ± 16.25^b^	135.45 ± 16.52^b^	134.64 ± 16.29^b^	92.36 ± 16.78^a^
*L. acidophilus*	QRR	151.26 ± 5.21^a^	148.59 ± 3.13^a^	151.29 ± 0.39^a^	148.28 ± 0.98^a^	149.71 ± 0.66^a^
	Rutin	16.13 ± 0.57^bc^	15.06 ± 0.38^a^^b^	16.90 ± 0.04^a^	14.63 ± 0.50^a^^b^	14.40 ± 0.24^a^
	Quercetin	<LOQ	<LOQ	<LOQ	<LOQ	0.53 ± 0.02^a^
	(+)-Catechin	6.55 ± 0.70^a^	5.71 ± 0.38^a^	5.35 ± 0.44^a^	4.80 ± 0.31^a^	4.65 ± 0.32^a^
	Gallic acid	2.70 ± 0.00^a^	2.67 ± 0.01^a^	2.79 ± 0.14^a^	2.83 ± 0.05^a^	2.96 ± 0.14^a^
	Ferulic acid	5.95 ± 0.00^a^	5.86 ± 0.09^a^	6.25 ± 0.02^b^	5.85 ± 0.05^a^	5.79 ± 0.01^a^
	Total	183.53 ± 5.53^a^	178.53 ± 3.23^a^	183.22 ± 1.43^a^	176.95 ± 1.90^a^	178.04 ± 0.86^a^

a *Values with no letter in common in the same line are significantly different (p < 0.05). QRR, quercetin-3-O-rutinose-7-O-α-L-rhamnoside; LOQ, limit of quantification; LOQ value was 0.01 mg/g*.

On the other hand, *B. uniformis* fermentation led to a degradation of (+)-catechin by 40.84% over 36 h of fermentation. However, (+)-catechin content in the supernatant of *B. uniformis* culture at 48 h was 20.80% higher than that at 36 h. After 48 h of fermentation, the content of (+)-catechin in *L. rhamnosus* culture was increased by 1.12 times as compared with the initial contents, while a descending trend was observed in *L. plantarum* cultures. Compared with the initial value, gallic acid content in the supernatant of *B. uniformis* culture at 48 h was significantly decreased. After 48 h of fermentation, the amounts of ferulic acid in *B. uniformis, L. rhamnosus*, and *L. plantarum* cultures were significantly decreased by 39.36, 34.98, and 23.91%, respectively, relative to the corresponding initial values. It was observed that the contents of the six aforementioned phenolic compounds in the *B. thetaiotaomicron* and *L. acidophilus* cultures showed no significant difference during fermentation, even though a slight range of fluctuation occurred (Figure 3).

### Effects of Single Microbial Fermentation on the Phenolic Composition

Only the supernatants of *B. uniformis, L. rhamnosus*, and *L. plantarum* cultures were subjected to UHPLC-ESI-MS/MS analysis in negative ionization mode and following experiments, as *B. thetaiotaomicron* and *L. acidophilus* exhibited no marked effects on LPP. According to the peak area, a total of 16 phenolic compounds were identified in all the samples (Table 2).

**Table 2:** Metabolites of lychee pulp phenolics in microbial cultures over 48 h fermentation^a^.

**No**.	**Compound**	**Molecular weight (Da)**	**Molecular formula**	**[M–H]^**−**^(Da)**	***Δm/z* (ppm)**	**ESI^**−**^major fragment ions (m/z)**	**Detection in sample**			
							**LPP-Un**	**LPP-*B. u***	**LPP-*L. r***	**LPP-*L. p***
1	Procyanidin B2	578	C_30_H_26_O_12_	576.99303	0.2	451; 425; 289	Y	N	Y	Y
2	Isorhamnetin-3-*O*-rhamnosylrutinoside	770	C_34_H_42_O_20_	769.21866	0.1	623; 315; 300; 271	Y	N	Y	Y
3	Isorhamnetin-3-*O*-rutinoside	624	C_28_H_32_O_16_	623.15981	0.2	315; 300; 299; 243	Y	N	Y	Y
4	Quercetin-3-*O*-rutinose-7-*O*-α-L-rhamnoside	756	C_33_H_40_O_20_	755.20284	0.0	489; 301; 300; 271; 179	Y	Y	Y	Y
5	Rutin	610	C_27_H_30_O_16_	609.14457	0.4	271; 343; 301; 179	Y	Y	Y	Y
6	Quercetin	302	C_15_H_10_O_7_	301.01383	0.4	273; 179; 151	N	Y	Y	Y
7	Catechin	290	C_15_H_14_O_6_	289.07123	1.3	271; 203; 109	Y	Y	Y	Y
8	Isorhamnetin	316	C_16_H_12_O_7_	315.05024	0.6	300; 283; 271; 151	N	Y	N	Y
9	Kaempferol	286	C_15_H_10_O_6_	285.24319	−0.3	239; 59	N	Y	N	N
10	Alphitonin	304	C_15_H_12_O_7_	303.04735	1.9	285; 241	N	N	Y	N
11	Ferulic acid	194	C_10_H_10_O_4_	193.05191	0.3	178; 149	Y	Y	Y	Y
12	Gallic acid	170	C_7_H_6_O_5_	169.01220	1.1	125; 79; 69	Y	Y	Y	Y
13	Caffeic acid	180	C_9_H_8_O_4_	179.05598	1.0	135	Y	Y	Y	Y
14	Dihydroxybenzaldehyde rhamnose	300	C_13_H_16_O_8_	299.02752	0.2	283; 267;193; 153	N	N	N	Y
15	Unkonwn	460	C_20_H_28_O_12_	459.27968	−0.3	443; 401; 375; 309	N	N	N	Y
16	Hydroxychromone	162	C_9_H_6_O_3_	161.06287	−0.9	145; 121	N	Y	N	N

a *LPP-Un, the unfermented LPP; LPP-B. u, the LPP fermented by B. uniformis for 48 h; LPP-L. r, the LPP fermented by L. rhamnosus for 48 h; LPP-L. p, the LPP fermented by L. plantarum for 48 h. Y represents that the phenolic compound detected in the sample; N represents that the phenolic compound was undetected in the sample*.

**Figure 4:**
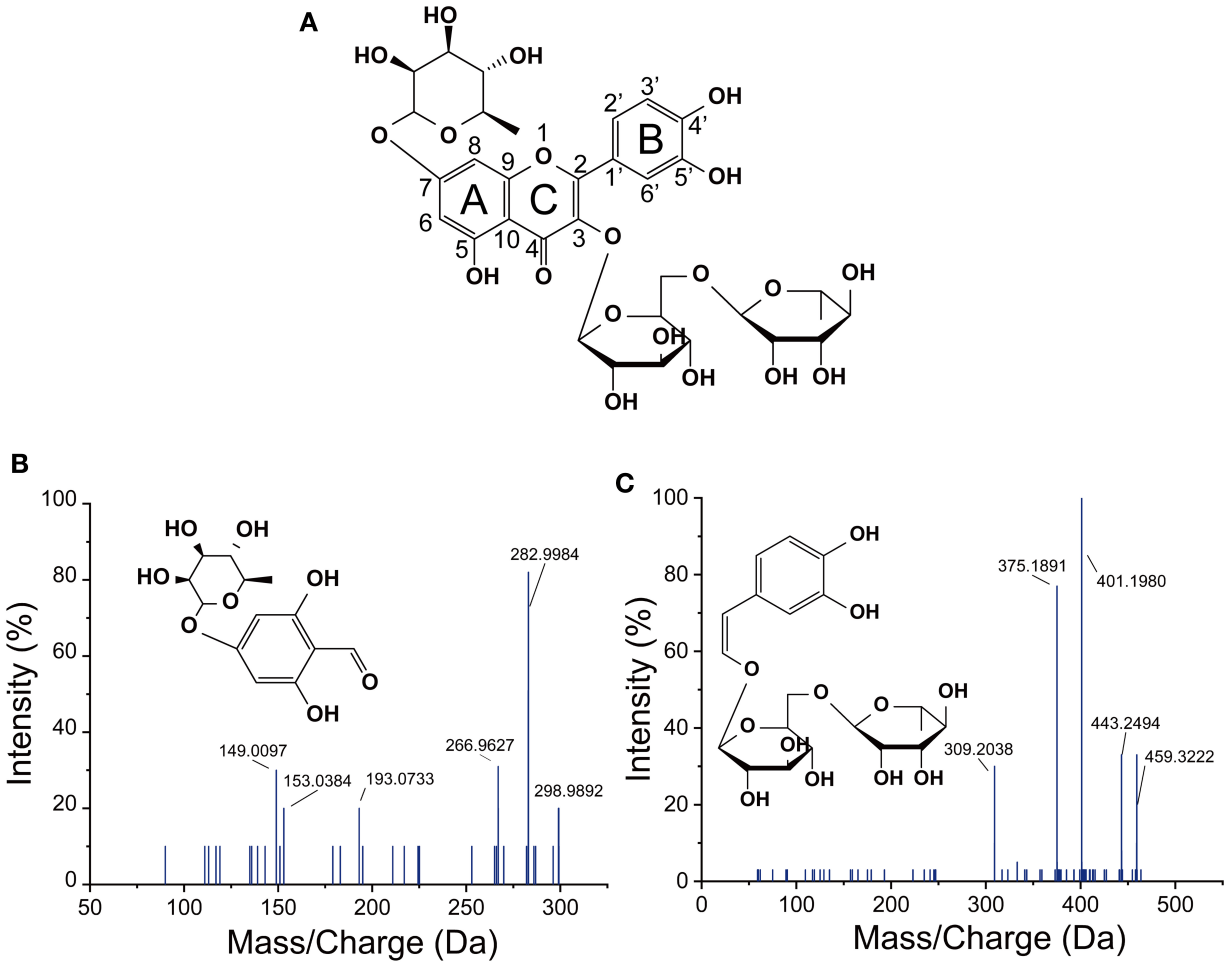
Structure of quercetin-3-*O*-rutinose-7-*O*-α-L-rhamnoside **(A)** and negative MS spectra **(B,C)** of two new-synthesized substances in *L. plantarum* culture by fission of C-ring of quercetin-3-*O*-rutinose-7-*O*-α-L-rhamnoside.

The mass spectrum of compound 1 showed a deprotonated molecular [M-H]^**−**^ ion at *m/z* 576.99303 and mass fragments in the MS^2^ spectrum at *m/z* 289, corresponding to the loss of procyanidin monomers (catechin) from the precursor ion (20). Compound 1 was identified as procyanidin B2. Compounds 2 and 3 gave an [M–H]^**−**^ ions at *m/z* 769.21866 and 623.15981, respectively. The mass fragments at *m/z* 315 (isorhamnetin) and 300 (rhamnetin) were found in their MS^2^ spectra, which were further confirmed as isorhamnetin-3-*O*-rhamnosylrutinoside (compound 2) and isorhamnetin-3-*O*-rutinoside (compound 3) (19), respectively. Quercetin-3-*O*-rutinose-7-*O*-α-L-rhamnoside (compound 4, MW = 756 Da) was detected with [M-H]^**−**^ at *m/z* 755.20284 and fragments in the MS^2^ at m/z 489 (fission of rutinoside), 301 (quercetin), and 300 (loss of rutinoside and rhamnose), whose chemical structure have been identified (Figure 4A) (5). Compound 5 with a deprotonated molecular [M-H]^**−**^ ion at *m/z* 609.14457, was highly consistent with quercetin-3-*O*-rutinose-7-*O*-α-L-rhamnoside in mass fragments in MS^2^ and was identified as rutin. Characteristic MS^2^ fragments at *m/z* 273 [M–CO]^**−**^, 179 (retro-Diels Alder reaction), and 151, indicated that the compound 6 was quercetin (21). Compound 7 with the deprotonated molecular [M–H]^**−**^ ion at *m/z* 289.07123 was identified as catechin, and the MS^2^ fragments at *m/z* 271(loss of H_2_O) and 203 (loss of five hydroxyl groups) reaffirm this structure (22). Compound 8 was identified as isorhamnetin, which gave an [M–H]^**−**^ ion at *m/z* 315.05024. The fragment ions at *m/z* 300 (loss of methyl) and 283 (loss of methyl and hydroxyl) and also *m/z* 151 reaffirm this structure (23). The fragment ions of compound 9 at *m/z* 257 (loss of -CO) and *m/z* 151 confirmed this compound as kaempferol (24). The fragment ions of compound 10 with [M–H]^**−**^ at *m/z* 303.04735, was similar to those (including *m/z* 285, 241, and 171) of alphitonin (8). Some phenolic acids, including ferulic acid (compound 11), gallic acid (compound 12), and caffeic acid (compound 13), were also identified, as previously reported (23, 24). Compound 14 with [M–H]^**−**^ at *m/z* 299.00325 was tentatively identified as dihydroxybenzaldehyde rhamnose. According to the fragment ions at m/z 282 (loss of hydroxyl) and 153, this structure was inferred (Figure 4B). However, compound 15 showing an [M–H]^**−**^ ion at *m/z* 459.01106, was still unclear (Figure 4C). Compound 16 showed an [M–H]^**−**^ ion at *m/z* 161.06287 and the MS^2^ fragments (at *m/z* 145 and 121), in accordance with the previous values for hydroxychromone (25).

Compared with unfermented LPP, three original phenolic compounds (including procyanidin B2, isorhamnetin-3-*O*-rhamnosylrutinoside and isorhamnetin-3-*O*-rutinoside) were degraded by *B. uniformis*. After 48 h of fermentation with *B. uniformis*, four new substances (including quercetin, isorhamnetin, kaempferol, and hydroxychromone) were generated and identified. As for *Lactobacillus. spp*, the newly synthesized quercetin and alphitonin were detected in the *L. rhamnosus* culture, while three substances (including quercetin, dihydroxybenzaldehyde rhamnose, and the unknown compound 15) were detected in the *L. plantarum* culture over 48 h of fermentation.

### Effects of Single Microbial Fermentation on TPC and Antioxidant Activities

**Figure 5:**
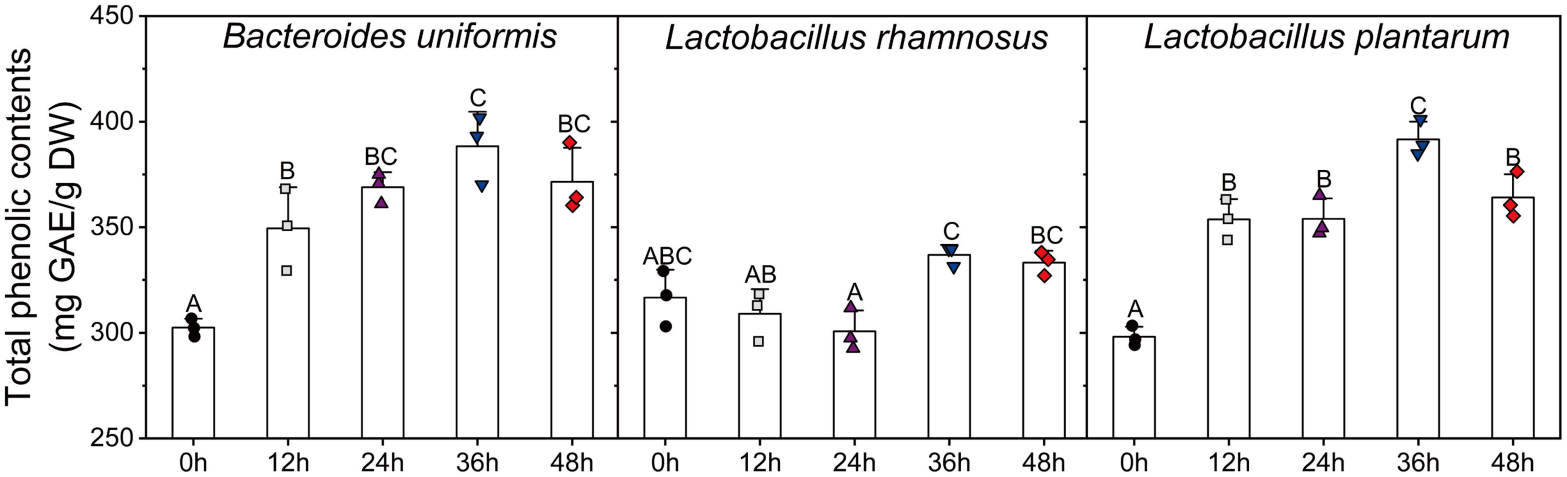
Changes in the total phenolic contents of the supernatants of different microbial cultures (*B. uniformis, L. rhamnosus* and *L. plantarum*) during fermentation with lychee pulp phenolics (LPP). Bars with no letter in common are significantly different (*p* < 0.05).

**Figure 6:**
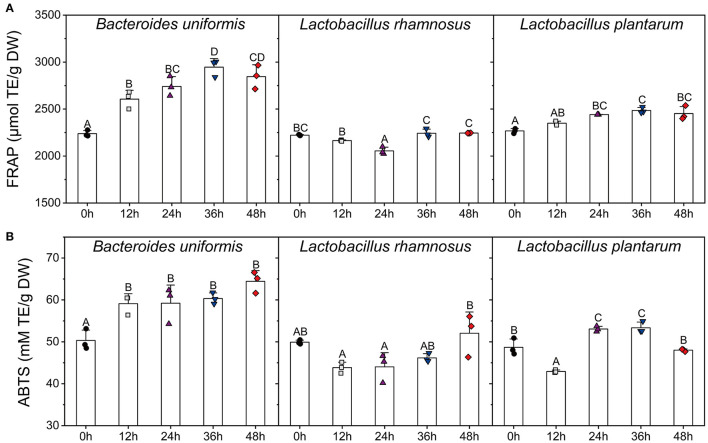
Changes in FFAR **(A)** and ABTS **(B)** scavenging capacity of the supernatants of different microbial cultures (*B. uniformis, L. rhamnosus*, and *L. plantarum*.) during fermentation with LPP. Bars with no letter in common are significantly different (*p* < 0.05). FRAP, ferric reducing antioxidant power.

The variations in TPC and antioxidant activities of individual cultures (*B. uniformis, L. rhamnosus*, and *L. plantarum*) were shown in Figures 5, 6. During *B. uniformis* fermentation, the TPC values of the supernatant at 12 h were 1.18 times of the initial values, and then reached a peak of 391.58 ± 6.90 mg GAE/g DW at 36 h. The TPC of the supernatant of *L. plantarum* culture at 36 h displayed an increase by 28%, relative to the initial values. However, the TPC value was slightly decreased by *L. rhamnosus* fermentation during the first 24 h and then increased to initial level.

After 36 h of fermentation, the FRAP values of both *B. uniformis and L. plantarum* cultures were increased to the highest levels during the fermentation. As for *L. rhamnosus*, the variation in FRAP antioxidant value was similar to TPC. The ABTS value of *B. uniformis* culture was significantly increased from 50.32 ± 2.03 to 59.12 ± 1.93 TE mM/g DW in the first 12 h, and then this value remained relatively stable until the end of fermentation. The highest ABTS activities occurred at 48 and 36 h for *L. rhamnosus* and *L. plantarum* cultures, respectively.

## Discussion

The interaction between phenolics and gut microbiota has been demonstrated in many prospective researches. Dietary phenolics exert a selective proliferative effect on gut microbiota and could simultaneously be catabolized (26). In this work, the prebiotic effects of LPP on *Bacteroides* and *Lactobacillus* were investigated and verified. Unprecedentedly, the specific microbial species (including *B. uniformis, L. rhamnosus*, and *L. plantarum*) which participated in LPP metabolism were ascertained. LPP metabolites from these three microbial species were identified individually, and the further analysis illustrated that various metabolic pathways of phenolic compounds were driven by different gut microbial species.

*Bacteroides* is a predominant microbial genus in human colon, whose abundance is closely associated with the intestinal metabolism and inflammatory level of the human host (27). It was reported that rutin could be converted to quercetin by *Bacteroides uniformis* and *Bacteroides ovatus* (28). In this work, not only flavonoid glycosides (e.g., quercetin-3-*O*-rutinose-7-*O*-α-L-rhamnoside and rutin) were deconjugated by *B. uniformis* resulting in an aglycone (quercetin) formation, but isorhamnetin glycoside derivatives were also hydrolyzed to form isorhamnetin. These were proved from the complete degradation of isorhamnetin-3-*O*-rhamnosylrutinoside and isorhamnetin-3-*O*-rutinoside, and the newly emergence of isorhamnetin in the *B. uniformis* culture after fermentation. In other words, *B. uniformis* is capable of releasing aglycones from various flavonoid glycosides of LPP. The improvement of biochemical environment (e.g., acid–base properties and carbon source) for microbial growth is one of probiotic mechanisms of phenolics (29). The turbidity and viable cell count of *B. uniformis* were both elevated after LPP supplementation, indicating the proliferative effect of LPP on this strain. Flavonoids in LPP extract contain phenolic hydroxyl groups, leading to a lower pH value of culture. This may provide a better biochemical environment for the growth of *B. uniformis*. On the contrary, the predominant flavonoids (e.g., quercetin-3-*O*-rutinose-7-*O*-α-L-rhamnoside, rutin, and isorhamnetin-3-*O*-rhamnosylrutinoside) in LPP are rhamnose derivatives. It has been pointed out that *Bacteroides* exerts an ability to catalyzing the fracture of rhamnoside bond and releasing rhamnose (28), which was in line with our results. Therefore, it was reasonable to infer that the increment in the viable cell count of *B. uniformis* was caused by the increased carbon source (rhamnose) from quercetin-3-*O*-rutinose-7-*O*-α-L-rhamnoside (QRR). The generation of hydroxychromone indicated that *B. uniformis* could breakdown the C2-C1' bond of quercetin. Although this metabolic pathway of quercetin has not been described, the fission of the C2-C1' bonds of hesperetin and naringenin by *Bifdobacterium longum* was reported (30). Notably, the demethoxylation of isorhamnetin and dehydroxylation of quercetin accounted for the generation of kaempferol in the *B. uniformis* culture. Du et al. (31) also demonstrated that isorhamnetin aglycon could be degraded to kaempferol by human intestinal flora. Whereas, the fact that *B. uniformis* is responsible for these activities has never been reported. In addition, although procyanidin B2 was not quantified in this work, its vanishment and the increment in the content of catechin were observed after *B. uniformis* fermentation. These results were caused by the fission of C-C bonds, which was in parallel to the previous findings of Tomas-Barberan and Espin (29). Although an increment in the turbidity of *B. thetaiotaomicron* culture was observed after LPP supplementation, the viable cell count of *B. thetaiotaomicron* was not increased. These results indicated that LPP showed no proliferative effect on *B. thetaiotaomicron in-vitro*. It has been reported that *B. thetaiotaomicron* participated in the degradation of flavonoids and other polymers by disrupting rhamnoside bonds (9, 32). Herein, all the phenolic compounds in LPP remained stable and could not been utilized by *B. thetaiotaomicron* during fermentation. Therefore, it was considered that the rhamnosidase from *B. thetaiotaomicron* displayed high specificity. A significant increment in *B. thetaiotaomicron* abundance was observed in mice after the treatment with LPP (13). The difference between *in-vitro* and *in-vivo* proliferative effect of LPP on *B. thetaiotaomicron* suggested the symbiotic relationship among various bacterial species in the colonic environment. This symbiotic relationship has attracted widely attention (33).

*Lactobacillus* is the dominant microbial unit in human intestine (34), and employed as probiotics in commercial products to augment the bioaccessibility of phenolics (35, 36). Liu et al. (37) investigated the effects of multiple *Lactobacillus* strains on the production of flavonoids, pointing out that *Lactobacillus* was involved in the transformation of flavonoids. It was noteworthy that rutin amounts showed a significant increase trend while QRR was catabolized by *L. rhamnosus* during the initial 36 h of fermentation. This result indicated that *L. rhamnosus* possessed the capacity to catabolize QRR into rutin. Particularly, with the synthesis of quercetin from flavonoid glycosides, alphitonin was also detected in the supernatant of *L. rhamnosus* culture. Previous research concerning with *in-vitro* catabolism of rutin by gut microbiota, reported that alphitonin was one of metabolites from its aglycone (8). Braune et al. (38) clarified that alphitonin was one of the intermediate products of quercetin that would thereafter be converted to gallic acid by *Eubacterium ramulus*. Therefore, it could be inferred that various gut microbial strains showed the identical metabolic pathways for the same substance. On the contrary, phenolic compounds have been deciphered as excellent free radical scavengers to prevent anaerobic probiotics from the redox stress (39). The supplementation of phenolics could alter the physiochemical properties of the surface of gut microbiota, leading to a higher level of negative charges, a reinforced hydrophobicity, which also contributed to the enhanced adhesion and biofilm formation of specific microbes (40, 41). The basal medium without LPP lacks of a certain kind of somatomedins, resulting in the decreased viable cell counts of *L. rhamnosus* after 48 h of fermentation. However, it was observed that LPP showed a positive effect on the viability of *L. rhamnosus*. Shubha et al. (41) found that a phenolic-riched woodfordia fruticosa extract stimulated the growth of *L. rhamnosus* by improving its intercellular adhesion and biofilm formation (41). Previous research also confirmed that flavonoids could strengthen the membrane fluidity of *L. rhamnosus* (42). These reasons may explain for the improvement of LPP in *L. rhamnosus* viability. Although the growth of *L. plantarum* and *L. acidophilus* was not improved by LPP, the viable cell counts of them are equivalent with those in the BLK group. Therefore, it could be deduced that LPP exhibits a positive effect on the proliferation of both *Bacteroides* and *Lactobacillus*.

Herein, it was observed that the increment in quercetin was significantly less than the reduction of QRR in *L. plantarum* culture after fermentation and no new peaks were found at 280 nm in the chromatogram. This result indicated that QRR was catabolized into some metabolites which were not belong to flavonoids. According to the peak areas detected by UHPLC-ESI-MS/MS, two new substances were synthesized by the fermentation with *L. plantarum*, with deprotonated molecule [M–H]^**−**^ ion at *m/z* 299.02752 and 459.27968, respectively. According to mass fragmentation pattern, the molecule would loss a hydrogen ion in the negative ion mode. The C-ring fissions of QRR at C2-O1 and C3-C4 bonds resulted in the generation of C_13_H_15_${\text{O}}_{8}^{-}$ and C_20_H_27_${\text{O}}_{12}^{-}$, corresponding to the aforedescribed deprotonated molecular [M–H]- ion accidently. Besides, the MS_2_ fragments also elucidated the newly generated substance with deprotonated molecular [M–H]^**−**^ ion at *m/z* 299.02752 contained rhamnose. Therefore, our results suggested that the pivotal metabolic way of QRR appeared to the direct fission of C-ring. In the wine model, the C-ring of anthocyanin glucosides and quercetin glycosides was broken by *Oenococcus oeni* and *L. plantarum*, resulting in the formation of phenolic acids (43). Besides, *Lactobacillus* involved in the C-ring fission of flavan-3-ols at 1- and 4- positions (44). Jujube juice incubated with *L. plantarum* presented an increase in the content of phenolic acids and a reduction in that of flavonoid glycosides (36), demonstrating that *L. plantarum* could synthesize enzymes to catalyze the fission of C-ring of flavonoids.

**Figure 7:**
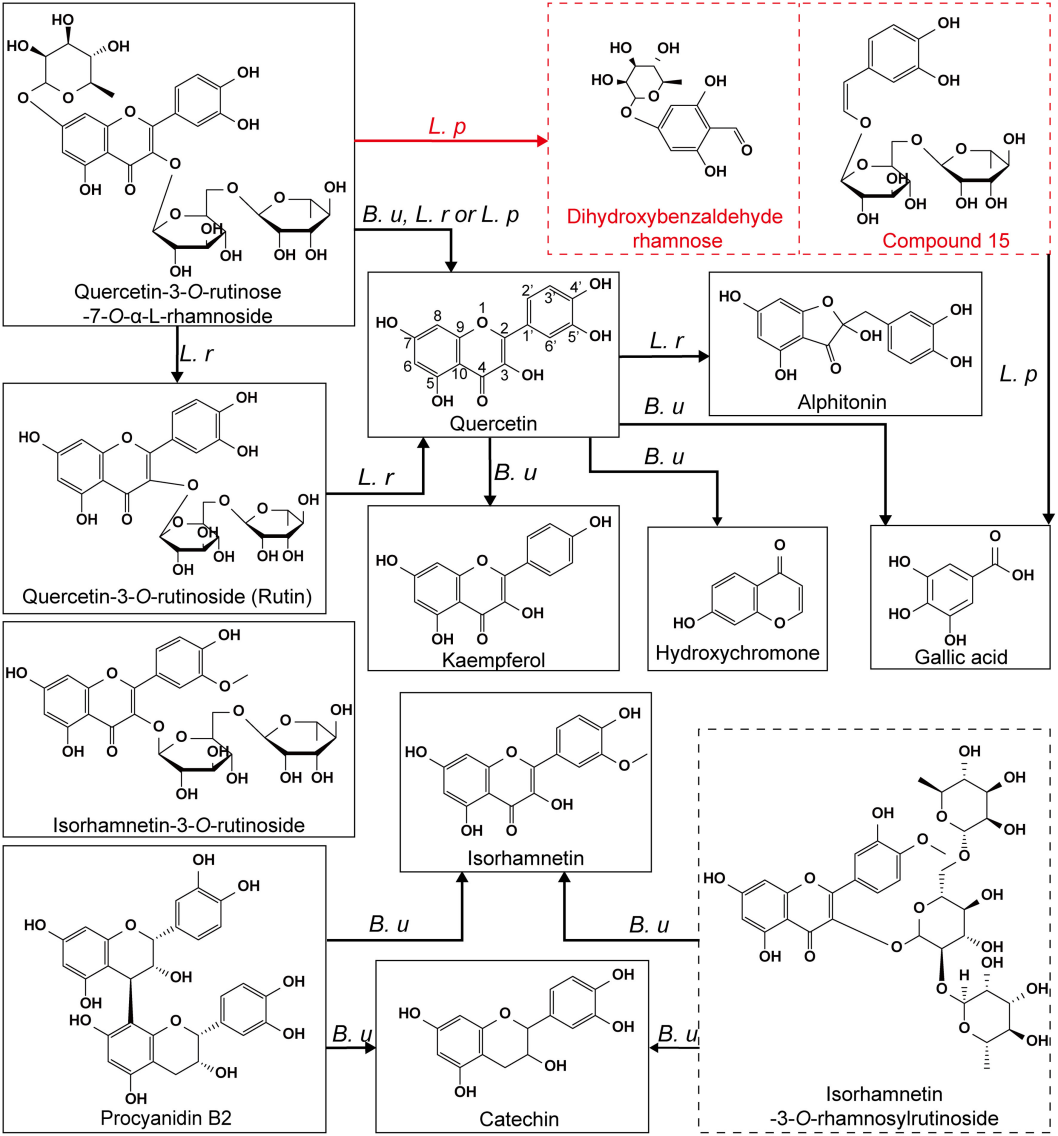
The proposed metabolic pathways of phenolic compounds in LPP by *B. uniformis, L. rhamnosus* and *L. plantarum*. The compounds framed with a dashed line represented the metabolites that were not verified. *B. u*, transformation occurred in the *B. uniformis* culture; *L. r*, transformation occurred in the *L. rhamnosus* culture; *L. p*, transformation occurred in the *L. plantarum* culture.

Therefore, it was deduced that QRR could be catabolized by the fission of C-ring directly with the presence of *L. plantarum*. Based on these results, the proposed metabolic pathways of phenolic compounds in LPP were generalized in Figure 7. Due to the complexity of metabolites, the precise chemical structures of these two compounds remained unclear. To further elucidate their chemical formulas, future studies are to separate and purify these substances, then subjecting to nuclear magnetic resonance spectroscopy analysis.

As Wu et al. (45) reported, phenolics could be catabolized into smaller units or metabolites which exerted higher bioavailability and bioactivity than their precursors. Among all the monocultures, the total phenolic content of LPP was largest enhanced by *B. uniformis* fermentation. Using correlation analysis, a linear relationship was established between TPC and FRAP (*R*^2^ = 0.715, *p* < 0.05), and between TPC and ABTS (*R*^2^ = 0.659, *p* < 0.05), suggesting that the antioxidant capacity was closely correlated with phenolic contents in the fermentation system. Combining HPLC-DAD assay, it was reasonable to infer that the decreased TPC value and antioxidant activity in *L. rhamnosus* fermentation were caused by the massive conversion of water-soluble QRR into insoluble substances, such as rutin and quercetin.

## Conclusion

The specific microbial species that participated in the metabolism of LPP were ascertained, including *B. uniformis, L. rhamnosus*, and *L. plantarum*. *B. uniformis* possessed a special capacity in catabolizing isorhamnetin glycoside (e.g., isorhamnetin-3-*O*-rhamnosylrutinoside and isorhamnetin-3-*O*-rutinoside) and quercetin glycoside (e.g., quercetin-3-*O*-rutinose-7-*O*-α-L-rhamnoside and rutin) into their corresponding aglycone. During *B. uniformis* fermentation with LPP, kaempferol was synthesized by the demethylation of isorhamnetin and dehydroxylation of quercetin, while procyanidin B2 was catabolized into catechin. The rhamnoside linkage of quercetin-3-*O*-rutinose-7-*O*-α-L-rhamnoside (QRR) was hydrolyzed by *L. rhamnosus*, resulting in the increased content of rutin. Quercetin, the aglycone of rutin and QRR was subsequently catabolized to alphitonin by *L. rhamnosus*. Notably, it was newly found that the pivotal metabolic pathways of quercetin-3-*O*-rutinose-7-*O*-α-L-rhamnoside by *L. plantarum* could be the direct fission of C-ring. Fermentation with *B. uniformis* could be used as a functional food to reinforce antioxidant activity of flavonoids.

## Data Availability Statement

The original contributions presented in the study are included in the article/Supplementary Material, further inquiries can be directed to the corresponding authors.

## Author Contributions

All authors listed have made a substantial, direct, and intellectual contribution to the work and approved it for publication.

## Funding

This study was supported by the Zhejiang Provincial Top Discipline of Biological Engineering (Grant No. KF2021005), the Science and Technology Program of Guangzhou (Grant No. 202102010451), the Guangdong Province's Key Research and Development Program (Grant No. 2020B0202080003), and the Agricultural Competitive Industry Discipline Team Building Project of Guangdong Academy of Agricultural Sciences (Grant No. 202108TD).

## Conflict of Interest

The authors declare that the research was conducted in the absence of any commercial or financial relationships that could be construed as a potential conflictof interest.

## Publisher's Note

All claims expressed in this article are solely those of the authors and do not necessarily represent those of their affiliated organizations, or those of the publisher, the editors and the reviewers. Any product that may be evaluated in this article, or claim that may be made by its manufacturer, is not guaranteed or endorsed by the publisher.
