# Supplementary material for: Novel Catabolic Pathway of Quercetin-3-O-Rutinose-7-O-α-L-Rhamnoside by Lactobacillus plantarum GDMCC 1.140: The Direct Fission of C-Ring
Source: Front Nutr. 2022 Mar 16;9:849439. doi: 10.3389/fnut.2022.849439 (PMC8966130; doi:10.3389/fnut.2022.849439)
Supplement: Supplementary file 1 [file Data_Sheet_1.docx]

***Supplementary Material***

# Supplementary Figures and Tables


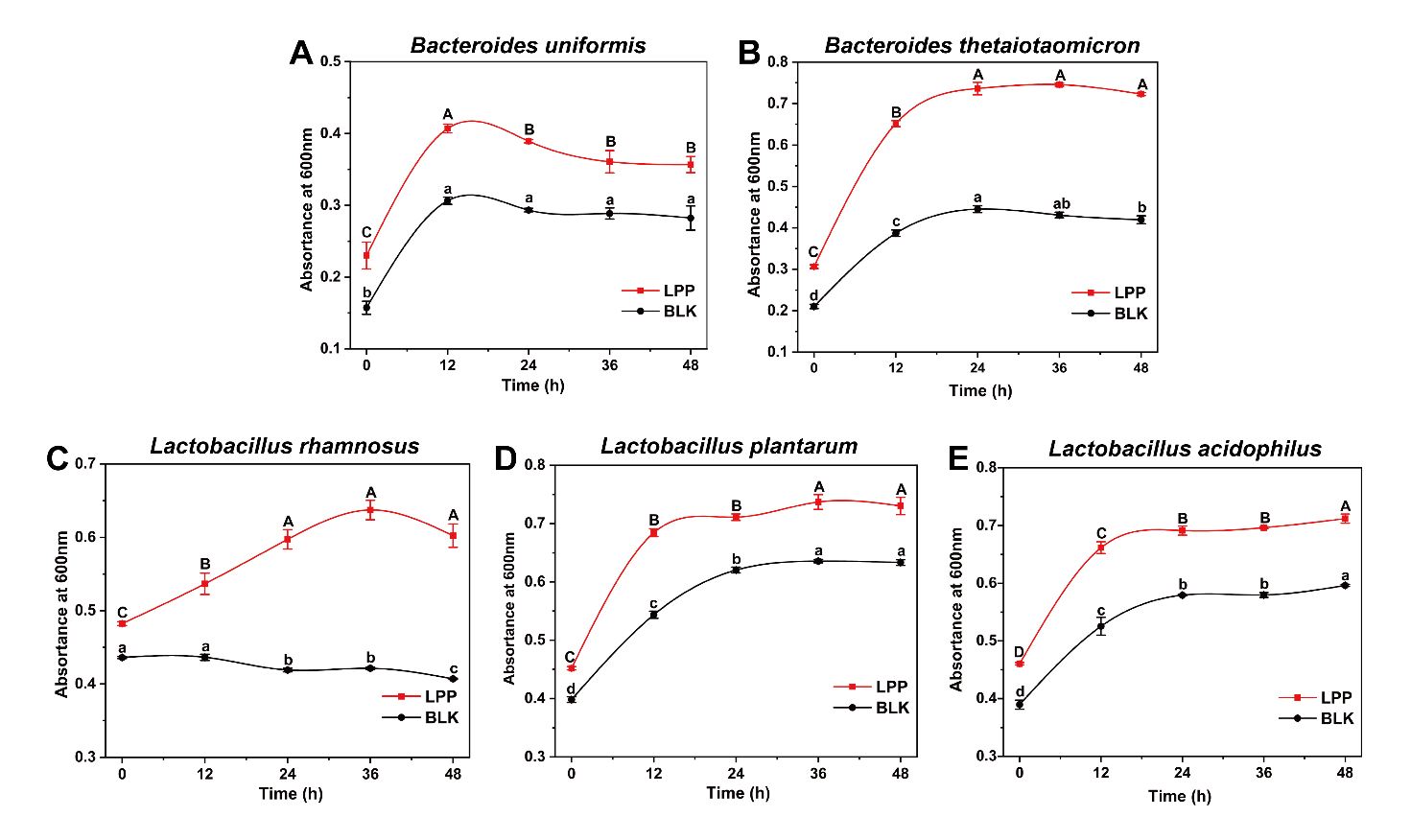


**Supplementary Figure 1.** Changes in the OD_600_ values (turbidity) of five microbial cultures during fermentation with lychee pulp phenolics (LPP). BLK group was fermented with PBS solution. Bars with no letter in common are significantly different (*p* < 0.05).


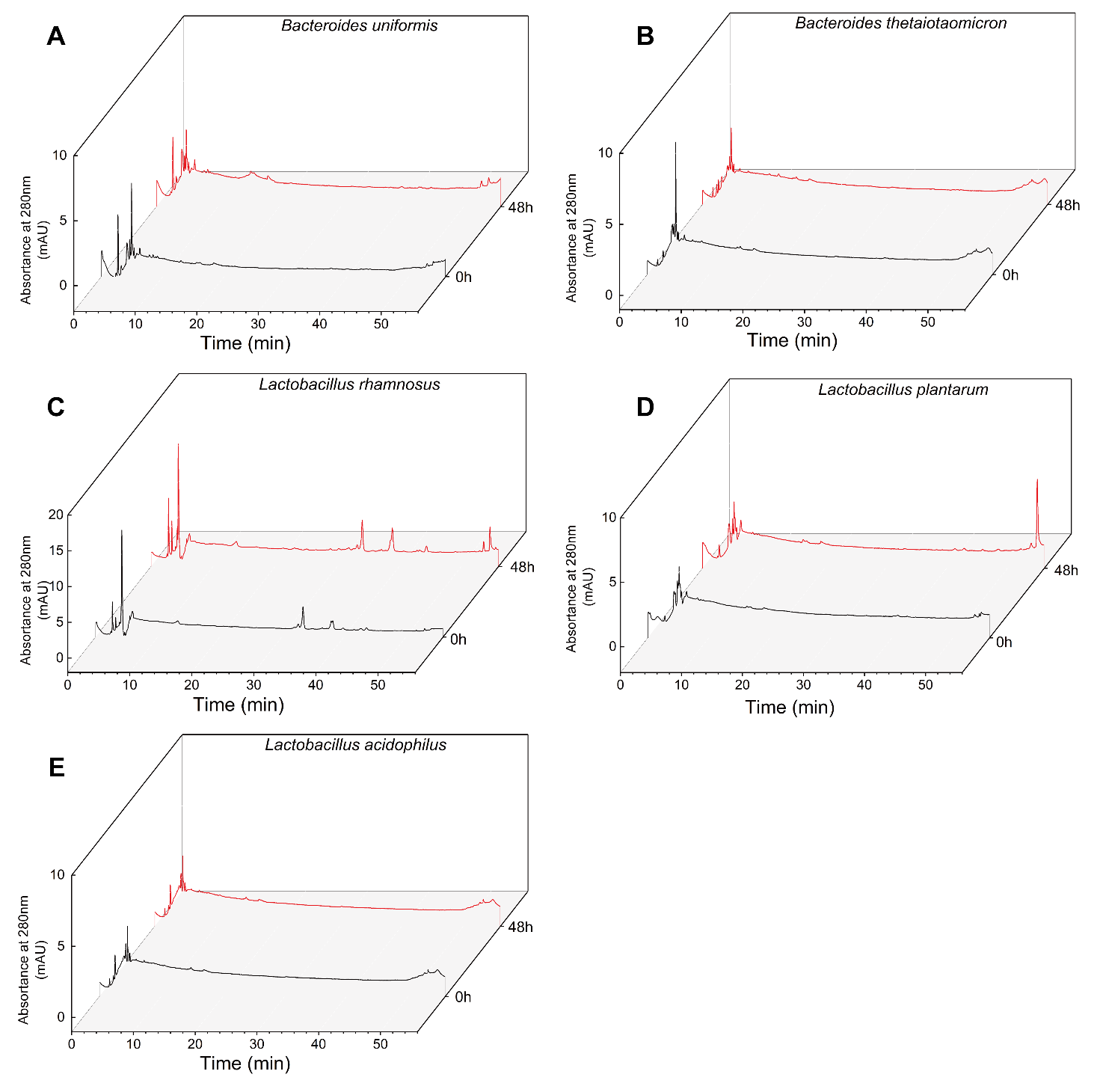


**Supplementary Figure 2.** Chromatograms of the residues of five gut microbial cultures at 0 and 48 h of fermentation with lychee pulp phenolics at 280 nm.

**Supplementary Table 1.** Phenolic contents of the residues of five microbial cultures during fermentation ^a^.

| Groups | Compound  (mg/g) | Fermentation times | | | | |
| --- | --- | --- | --- | --- | --- | --- |
|  |  | 0 h | 12 h | 24 h | 36 h | 48 h |
| *B. uniformis* | QRR | <LOQ | <LOQ | <LOQ | <LOQ | <LOQ |
|  | Rutin | <LOQ | <LOQ | <LOQ | <LOQ | <LOQ |
|  | Quercetin | <LOQ | <LOQ | <LOQ | <LOQ | <LOQ |
|  | (+)-Catechin | <LOQ | <LOQ | <LOQ | <LOQ | <LOQ |
|  | Gallic acid | <LOQ | <LOQ | <LOQ | <LOQ | <LOQ |
|  | Ferulic acid | <LOQ | <LOQ | <LOQ | <LOQ | <LOQ |
| *B. thetaiotao*  *micron* | QRR | <LOQ | <LOQ | <LOQ | <LOQ | <LOQ |
|  | Rutin | <LOQ | <LOQ | <LOQ | <LOQ | <LOQ |
|  | Quercetin | <LOQ | <LOQ | <LOQ | <LOQ | <LOQ |
|  | (+)-Catechin | <LOQ | <LOQ | <LOQ | <LOQ | <LOQ |
|  | Gallic acid | <LOQ | <LOQ | <LOQ | <LOQ | <LOQ |
|  | Ferulic acid | <LOQ | <LOQ | <LOQ | <LOQ | <LOQ |
| *L. rhamnosus* | QRR | <LOQ | 1.90±0.01^C^ | 2.05±0.02^D^ | 2.45±0.03^E^ | 1.25±0.01^B^ |
|  | Rutin | <LOQ | 0.47±0.01^A^ | 0.57±0.01^B^ | 0.66±0.01^C^ | 0.60±0.02^C^ |
|  | Quercetin | <LOQ | 0.12±0.00^A^ | 0.15±0.01^A^ | 0.21±0.02^B^ | 0.25±0.00^C^ |
|  | (+)-Catechin | <LOQ | <LOQ | <LOQ | <LOQ | <LOQ |
|  | Gallic acid | <LOQ | <LOQ | <LOQ | <LOQ | <LOQ |
|  | Ferulic acid | <LOQ | <LOQ | <LOQ | <LOQ | <LOQ |
| *L. plantarum* | QRR | <LOQ | <LOQ | <LOQ | <LOQ | <LOQ |
|  | Rutin | <LOQ | <LOQ | <LOQ | <LOQ | <LOQ |
|  | Quercetin | <LOQ | <LOQ | <LOQ | 0.40±0.01^A^ | 0.77±0.05^B^ |
|  | (+)-Catechin | <LOQ | <LOQ | <LOQ | <LOQ | <LOQ |
|  | Gallic acid | <LOQ | <LOQ | <LOQ | <LOQ | <LOQ |
|  | Ferulic acid | <LOQ | <LOQ | <LOQ | <LOQ | <LOQ |
| *L**. acidophilus* | QRR | <LOQ | <LOQ | <LOQ | <LOQ | <LOQ |
|  | Rutin | <LOQ | <LOQ | <LOQ | <LOQ | <LOQ |
|  | Quercetin | <LOQ | <LOQ | <LOQ | <LOQ | <LOQ |
|  | (+)-Catechin | <LOQ | <LOQ | <LOQ | <LOQ | <LOQ |
|  | Gallic acid | <LOQ | <LOQ | <LOQ | <LOQ | <LOQ |
|  | Ferulic acid | <LOQ | <LOQ | <LOQ | <LOQ | <LOQ |

^a^ Values with no letter in common in the same line are significantly different (*p* < 0.05). QRR, quercetin-3-*O*-rutinose-7-*O*-α-L-rhamnoside; LOQ, limit of quantification; LOQ value was 0.01mg/g.
